# Patterns and socioeconomic influences of tobacco exposure in tobacco cultivating rural areas of Yunnan Province, China

**DOI:** 10.1186/1471-2458-12-842

**Published:** 2012-10-04

**Authors:** Le Cai, Xinan Wu, Abhinav Goyal, Yuntao Han, Wenlong Cui, Xia Xiao, Jianhui He, Keying Zhao, Ying Song, Feng Jiao

**Affiliations:** 1 Cheng Gong New City, School of Public Health, Kunming Medical University, 1168 Yu Hua Street Chun Rong Road, Kunming, 650500, China; 2 Emory University, Atlanta, GA, 30322, USA

**Keywords:** Smoking, Exposure to secondhand smoke, Socioeconomic status, China

## Abstract

**Background:**

This study describes the patterns and socioeconomic influences of tobacco use among adults in tobacco-cultivating regions of rural southwest China.

**Methods:**

A cross-sectional survey was conducted in 8681 adults aged ≥18 years in rural areas of Yunnan Province, China from 2010 to 2011. A standardized questionnaire was administered to obtain data about participants’ demographic characteristics, individual socioeconomic status, ethnicity, self-reported smoking habits, and exposure to secondhand smoke (SHS). The socioeconomic predictors of current smoking, nicotine addiction, and SHS exposure were analyzed using multivariate logistic regression.

**Results:**

The prevalence rates of tobacco use were much higher in men compared with women (current smoking 68.5% vs. 1.3%; and nicotine dependence 85.2% vs. 72.7%). However, the rate of SHS exposure was higher in women compared with men (76.6% vs. 70.5%). Tobacco farmers had higher prevalence rates of current smoking, nicotine dependence, and SHS exposure compared with participants not engaged in tobacco farming (P<0.01). Most tobacco users (84.5%) reported initiating smoking during adolescence. A total of 81.1% of smokers smoked in public places, and 77.6% smoked in workplaces. Individuals belonging to an ethnic minority had a lower probability of SHS exposure and nicotine dependence. Individual educational level was found to be inversely associated with the prevalence of current smoking, exposure to SHS, and nicotine dependence. Higher annual household income was associated with a greater risk of nicotine dependence.

**Conclusions:**

This study suggests that tobacco control efforts in rural southwest China must be tailored to address tobacco-cultivating status and socioeconomic factors.

## Background

The health effects of smoking and exposure to secondhand smoke (SHS) are major global public health issues. The World Health Organization reported that tobacco use in the 20th century resulted in 100 million deaths worldwide [1], and this number is projected to increase to 1 billion deaths in the 21^st^ century. China is the most important battleground in the struggle against tobacco use globally, and it is both the world’s largest producer and consumer of tobacco products. China is home to an estimated 301 million current smokers in 2010, and the rising trends in tobacco consumption are alarming [2]. The 2002 national survey reported that 51.9% of non-smokers in China were regularly exposed to SHS [3]. Correspondingly, China also experiences a high incidence of tobacco-related diseases [4]. A previous Chinese study indicates that a total of 673,000 deaths in Chinese adults were attributable to smoking in 2005 [5], with cancer, cardiovascular disease, and respiratory disease accounting for most of these deaths. Smoking is highly prevalent, and its impact on premature death has become a major concern in China.

Only through understanding the major factors that contribute to smoking behavior can we hope to design and execute effective tobacco control policies. There is growing evidence that patterns of smoking are related to age, sex, socioeconomic status (SES), and health risk behavior [6-8]. People who are less educated, who earn less, and who are unemployed show higher prevalence rates of smoking in developed countries. An inverse relationship between educational attainment and the risks of environmental tobacco exposure was also identified in previous Chinese and Korean studies [9,10]. In China, several studies have examined the relationship between smoking and SES variables in the general adult Chinese population [10-12], and show an inverse association between education and smoking. However, little data are available concerning the associations between SES variables and tobacco use in the Chinese tobacco-cultivating adult population. It is crucial that we understand the socioeconomic factors related to tobacco use and identify target populations for tobacco control. Furthermore, the epidemiological studies conducted in China have focused mostly on urban areas or national-level data, but the literature is still sparse in tobacco-cultivating rural areas, where the rate of tobacco consumption is the highest.

The aim of this study is to describe the patterns and socioeconomic influences of tobacco use among southwest China’s tobacco-cultivating rural adult population from 2010 to 2011.

## Methods

### Study region

This study used a community-based cross-sectional survey and was conducted in two tobacco-cultivating rural areas of Yunnan Province in southwest China. In 2010, Yunnan Province had 129 official counties and a total population of 45.9 million people. It is a relatively undeveloped province, and is a production and consumption hub for tobacco products. Roughly 70% of Yunnan Province’s annual tax revenue is collected from the tobacco industry, and tobacco is cultivated in 45 of the province’s counties [10]. Yunnan also has the country’s largest concentration of ethnic minorities, with 25 of China’s 56 state-recognized ethnicities living in the region. For decades, the regional government of Yunnan has depended on the economic engine of tobacco. As a result, the strong enforcement of existing tobacco control laws is not fully supported by the Yunnan government. The progress on tobacco control is not moving quickly in this region. More recently, Yunnan government implemented the anti-tobacco policy to prohibit smoking in public institutions including hospitals, healthcare centers and schools.

### Sampling

Multi-stage stratified random sampling was used to select the study sample. In the first stage, all of Yunnan Provinces’ tobaccos cultivating counties were classified into two groups according to wealth distribution (per capita Gross Domestic Product): economically advantaged (<$600) and economically disadvantaged (≥$600) based on the 2010 Yunnan Statistical Yearbook [13]. In order to guarantee the representativeness of each group, one county was randomly selected from each of these two economic groups. In the second stage, all township districts were selected for study in the two chosen counties, for a total of 21 townships. In the third stage, three villages were chosen by probability proportional to size (PPS) from each of the 21 townships. In the final stage, in each selected village, we first obtained a list of names of individuals aged ≥ 18 years from the village committee, and then simple random sampling was done with random number tables to select the individuals from this village.

### Data collection and measurement

Ten 5th-year medical students and twenty master degree students from Kunming Medical University were selected and extensively trained as interviewers for data collection. A workshop was held before the fieldwork to teach interviewers how to administer the screening questionnaire, and how to take blood pressure and anthropometric measurements. Each participant was given a full explanation of the research purpose and invited to participate; signed informed consent; and was personally interviewed by one of the interviewers using a pre-tested structured questionnaire. The questionnaire asked for demographic characteristics, individual SES, ethnicity, household income, educational level, lifestyle factors such as alcohol consumption, self-reported smoking habits, exposure to SHS at home or work, and tobacco use-related health status.

### Ethical approval

This study was approved by the Ethics Committee of Kunming Medical University.

### Definitions

Current smoker was defined as a person who currently smoked any kind of tobacco product on a daily basis at the time of the survey. Exposure to SHS was defined as non-smokers who reported exposure to environmental tobacco smoke at home or at work for a minimum of 15 minutes at least one day per week. The location of exposure to SHS was defined as in the past seven days, the locations where others have smoked in front of a current smoker. Current drinker was defined as a person who drank alcohol regularly on 12 or more days during the past 12 months. Self-reported current smokers were assessed for nicotine dependence using six questions from the Fagerstrom Test for Nicotine Dependence (FTND) [14]. The FTND has 6 items with an overall score ranging between 0–10. Very high dependence was defined as an FTND score ranging between 8–10; high dependence, a score between 6–7; medium dependence, a score equal to 5; low dependence, a score between 3–4; and very low dependence, a score between 0–2.

### Statistical analysis

Descriptive analysis techniques, chi-squared test, and multivariate logistic regression were used in this study. Yearly household income was classified into three categories: low (<$150 USD), medium ($150-600 USD), and high (>$600 USD). Categorical variables were presented as counts and percentages, and 95% confidence intervals (95% CI) for percentages were calculated according to the method described by Newcombe [15]. The sampling weights were calculated based on data from the year 2000 Yunnan Provincial Population Census and our sampling scheme. The prevalence of current smoking, nicotine dependence and exposure to SHS, however, were estimated based on weighted proportions. Chi-squared test was used to determine the percentage differences between men and women. Logistic regression was used to analyze the association between individual SES variables and the weighted prevalence of current smoking, intensity of smoking, exposure to SHS, and nicotine dependence. Associations were expressed as odds ratios and 95% CI. All statistical significance decisions were based on a two-tailed P value of <0.05. Due to large differences in smoking behavior between men and women, interactions were tested to determine whether SES variables varied by sex. All data analyses were conducted with R2.9.1 software [14].

## Results

A total of 8,800 individuals aged ≥18 years were invited to participate in this study. Of these, 8,681 agreed to participate (response rate = 98.6%).

As shown in Table 1, the study population included 4,068 males and 4,631 females, 22.3% of which belonged to an ethnic minority, and 77.7% of which were Han Chinese. The proportion of participants who engaged in tobacco cultivation was 28.1%. Male participants had higher educational level than female participants (P<0.05).

**Table 1:** Demographic characteristics of the study population

**Characteristics (%)**	**Male (n=4068)**	**Female (n=4613)**	**All (n=8681)**
Age			
18-34 years	1078 (26.5)	1099 (23.8)	2177 (25.1)
35-44 years	864 (21.2)	923 (20.0)	1787 (20.6)
45-54 years	702 (17.3)	850 (18.4)	1552 (17.9)
55-64 years	666 (16.4)	843 (18.3)	1509 (17.4)
≥65 years	758 (18.6)	898 (19.5)	1656 (19.1)
Tobacco cultivating			
Yes	1267 (31.1)	1176 (25.5)	2443 (28.1)
No	2801 (68.9)	3437 (74.5)	6238 (71.9)
Ethnicity			
Han	3226 (79.3)	3516 (76.2)	6742 (77.7)
Minorities	842 (20.7)	1097 (23.8)	1939 (22.3)
Level of education			
Primary (grade 1–6) or lower	3402 (83.6)*	4107 (89.0)*	7509 (86.5)
Middle ((grade 7–9) or higher	666 (16.4)	506 (11.0)	1172 (13.5)
Level of yearly household income^†^			
Low	856 (21.0)	1096 (23.8)	1952 (22.5)
Medium	2285 (56.2)	2701 (58.6)	4986 (57.4)
High	927 (22.8)	816 (17.7)	1743 (20.1)

* P<0.05 from chi-squared test for difference between sexes.

† Low income is < $150 USD; medium is $150-600 USD; and high income is >$600.

Table 2 describes weighted prevalence of current smokers, nicotine dependence, and exposure to SHS by age, sex and tobacco-cultivating status among southwest China’s rural adult population. The overall prevalence rates of current smokers and exposure to SHS were 33.7% and 74.2% respectively, and the rate of nicotine dependence among smokers was 84.8%. In general, men reported much higher rates of smoking and nicotine dependence than women (P<0.01), whereas women reported higher rate of exposure to SHS than men (P<0.05). The highest rates of current smokers, exposure to SHS, and nicotine dependence were found among individuals aged 35–44 years. Individuals who cultivated tobacco had higher prevalence rates of current smoking, exposure to SHS, and nicotine dependence than non-tobacco cultivating people (P<0.01).

**Table 2:** Weighted prevalence of current smoking, nicotine dependence, and secondhand smoke (SHS) by age, sex and status of tobacco cultivation

**Characteristics**	**Prevalence of current smokers**			**Prevalence of nicotine dependence among smokers**			**Prevalence of SHS among non-smokers**		
	**Men**	**Women**	**All**	**Men**	**Women**	**All**	**Men**	**Women**	**All**
	**% (95% CI)**	**% (95% CI)**	**% (95% CI)**	**% of smokers (95% CI)**	**% of smokers (95% CI)**	**% of smokers (95% CI)**	**% of non-smokers (95% CI)**	**% of non-smokers (95% CI)**	**% of non-smokers (95% CI)**
Age									
18-34 years	63.3 (60.3-66.0)	0.5 (0.1-0.8)	31.3 (29.5-33.9)	82.0 (79.8-85.5)	48.8 (9.45-90.6)	82.3 (58.6-86.0)	70.8 (65.6-74.6)	76.9 (74.9-79.9)	74.7 (73.4-77.7)
35-44 years	76.7 (73.7-79.3)	0.9 (0.2-1.0)	37.5 (35.1-40.6)	86.1 (83.6-88.8)	74.6 (30.1-95.4)	87.0 (64.2-90.3)	73.0 (65.7-78.0)	80.1 (78.1-83.2)	78.7 (76.7-81.5)
45-54 years	74.0 (70.9-77.3)	1.0 (0.6-2.1)	34.4 (31.8-36.5)	88.1 (84.2-89.9)	89.3 (56.5-98.0)	87.8 (78.3-96.2)	70.2 (63.7-76.9)	81.3 (77.9-84.3)	80.1 (76.4-82.4)
55-64 years	70.1 (66.1-73.0)	1.1 (0.4-1.6)	31.0 (28.9-33.6)	84.9 (81.8-88.3)	85.4 (48.7-97.4)	85.2 (68.2-94.7)	68.8 (59.1-74.0)	77.3 (73.1-79.9)	75.4 (71.3-77.7)
≥65 years	59.0 (56.1-63.1)	1.6 (1.0-2.8)	28.4 (26.1-30.4)	83.8 (80.7-87.4)	58.7 (35.8-80.2)	83.5 (67.1-83.5)	67.2 (65.9-76.0)	64.1 (62.0-68.3)	65.3 (64.1-69.4)
Tobacco cultivating									
Yes	78.0 (75.1-79.7)	1.7 (0.5-1.7)	43.0 (38.7-45.6)	89.1 (86.5-91.4)	89.2 (59.6-98.2)	89.0 (84.5-90.4)	82.3 (77.2-86.1)	86.0 (83.3-87.8)	85.1 (82.9-87.6)
No	63.9 (62.4-65.9)	0.7 (0.6-1.2)	28.9 (28.2-30.4)	82.7 (81.4-84.9)	67.1 (47.8-81.4)	82.8 (81.0-84.7)	66.2 (63.8-69.7)	72.1 (71.4-74.4)	70.6 (69.2-72.8)
All	68.5 (66.9-69.7)	1.3 (0.8-1.6)	33.7 (31.5-35.5)	85.2 (83.7-86.4)	72.7 (57.0-84.6)	84.8 (83.1-86.3)	70.5 (67.6-72.6)	76.6 (74.8-77.8)	74.2 (73.1-75.9)

Table 3 displays patterns of tobacco exposure among study participants. Filtered cigarette smoking was the most popular form of smoking tobacco, comprising 66.5% of all forms of tobacco consumed, followed by Hookah / Water pipe (57.7%). However, there were important differences in these findings by sex. Filtered cigarette smoking was the most popular form of smoking tobacco for men, whereas hookah / Water pipe was the most popular form of smoking tobacco for women. Age of smoking initiation occurred mostly during adolescence (84.5%). 51.9% of current smokers reported daily smoking of more than 10 cigarettes. A total of 6.8% of current smokers were highly nicotine addicted. In the 12 months before the survey, only 13.2% of smokers tried at least one attempt to quit smoking. In the past seven days, 81.1% of smokers smoked in public places (82.0% for men, and 16.2% for women); 77.6% smoked in workplace (78.3% for men, and 27.0% for women); 33.9% smoked in front of a pregnant woman (40.2% for men, and 11.3% for women); and 84.6% smoked in front of children while in indoor places (82.1% for men, and 74.1% for women).

**Table 3:** Patterns of tobacco exposure among current smokers in rural areas of Yunnan province, China

**Variables**	**Men (N=2780)**	**Women (N=37)**	**All (N=2817)**
	**n (%)**	**n (%)**	**n (%)**
Use of various forms of tobacco^+^			
Filtered cigarettes	1718 (61.8)	16 (43.2)	1734 (61.6)
Hookah / Water pipe	1447 (52.1)	22 (59.5)	1469 (52.1)
Pipe tobacco	137 (4.9)	5 (13.5)	142 (5.0)
Hand-rolled cigarettes	137 (4.9)	1 (2.7)	138 (4.9)
Unfiltered cigarettes	103 (3.7)	0 (0.0)	103 (3.7)
Chewing tobacco	31 (1.1)	0 (0.0)	31 (1.1)
Age of smoking initiation			
<=11 years	97 (3.5)	2 (5.4)	99 (3.5)
12-20 years	2257 (81.2)	26 (70.2)	2283 (81.0)
21-34 years	405 (14.6)	5 (13.5)	410 (14.6)
>=35 years	21 (0.8)	4 (10.8)	25 (0.9)
Previous quit attempts			
At least one attempt in previous 12 months	368 (13.2)*	3 (8.1)*	371 (13.2)
No attempt	2412 (86.8)	34 (91.9)	2446 (86.8)
Amount of smoking per day (cigarettes)			
<10	1323 (47.6)*	33 (89.2)*	1356 (48.1)
>=10	1457 (52.4)	4 (10.8)	1461 (51.9)
Nicotine dependence			
High or very high dependence (score 6–10)	189 (6.8)	2 (5.4)	191 (6.8)
Medium dependence (score 5)	225 (8.1)	3 (8.1)	228 (8.1)
Low or very low dependence (score less than 5)	2366 (85.1)	32 (86.5)	2398 (85.1)
In the past seven days, the conditions under which others have smoked in front of you			
Public spaces (schools, hospitals, etc.)	2280 (82.0)**	6 (16.2)**	2286 (81.1)
Workplace (labor field)	2176 (78.3)**	10 (27.0)**	2186 (77.6)
In front of a pregnant woman while in indoor places ^++^	331 (40.2)**	2 (11.3)**	351 (33.9)
In front of children while in indoor places ^+++^	1657 (82.1)	20 (74.1)	1677 (84.6)

* P<0.05 from chi-squared test for difference between sexes, ** P<0.01 from chi-squared test for difference between sexes, † The current smoker used more than one form of smoking tobacco, †† The denominator is the number of current smokers living with children at home, ††† The denominator is the number of current smokers living with pregnant woman at home.

We found no association between any SES indicators and the intensity of smoking. Table 4 shows the results of logistic regression for prevalence of current smokers, nicotine dependence, and exposure to SHS after adjusting for possible confounders. After adjusting for age, sex, tobacco cultivation, drinking status, and type of county, as well as further adjusting for other measures of SES, individuals who belong to an ethnic minority had a lower probability of nicotine dependence and of being exposed to SHS. Individuals with higher levels of education had a lower probability of current smoking, exposure to SHS, and nicotine dependence. Furthermore, higher yearly household incomes were found to be associated with a higher risk of nicotine dependence, but not with current smoking or SHS exposure. Individuals who cultivated tobacco had a higher probability of current smoking, exposure to SHS, and nicotine dependence. Although we tested for possible interactions, there were no significant interactions between sex and SES variables.

**Table 4:** Weighted Logistic regression coefficients for current smoking, nicotine dependence and exposure to secondhand smoke (SHS) by ethnicity and socioeconomic status

**Variables**	**Current smokers**		**Nicotine dependence**		**SHS**	
	**(N=8681)**		**(N=8681)**		**(N=8681)**	
	**Adjusted odds ratio†**	**95% CI**	**Adjusted odds ratio†**	**95% CI**	**Adjusted odds ratio†**	**95% CI**
Ethnicity (reference: Han)						
Minorities	0.93	(0.79, 1.10)	0.79**	(0.69, 0.95)	0.84**	(0.74, 0.95)
Level of education (reference: primary (grade 1–6) or lower)						
Middle (grade 7–9) or higher	0.63**	(0.51, 0.78)	0.56**	(0.46, 0.65)	0.51**	(0.43, 0.61)
Level of yearly household income (reference: Low)						
Medium	1.08	(0.97, 1.20)	1.16*	(1.04, 1.31)	1.06	(0.93, 1.11)
High	1.05	(0.93, 1.18)	1.11*	(1.02, 1.19)	1.00	(0.90, 1.06)
Tobacco cultivating (reference: no)						
Yes	3.18**	(2.77, 3.65)	2.42**	(2.12, 2.77)	1.35**	(1.17, 1.55)

* p<0.05, ** p<0.01, † adjusted for age, sex, and alcohol drinking.

## Discussion

The findings indicate a high prevalence of smoking, and a high prevalence of exposure to SHS among non-smokers. There was also a high rate of SHS exposure among current smokers in public places, and a low rate of attempting to quit smoking among current smokers in rural southwest China. Individual SES was found to be independently associated with the prevalence of current smoking, exposure to SHS, and nicotine dependence.

It has been estimated that worldwide, men smoke more than women. Our finding is in concordance with other studies [16]. In this study, the prevalence of smoking among men was greater than the prevalence rate observed in 2010 China Global Adults Tobacco Survey (GATS, 56.1%) [17], other parts of rural China (66.8%) [10], adults from western countries [7,8], and other Asian and African people [18-20]. Smoking has been very prevalent in the Chinese tobacco cultivating rural male population. In contrast to the high prevalence of smoking, our study reported a low prevalence of smokers who have previously attempted to quit smoking (18.2%), a rate that was much lower than reported by the 2010 China GATS survey (33.1%), in other cities of China (25.3%) [21], and in other western countries (40%) [22]. These data indicate that rural communities are in need of robust smoking cessation programs.

Several previous Chinese studies indicate that most Chinese smokers initiate smoking during adolescence, and the prevalence of adolescent smoking has been increasing rapidly in China [23,24]. Our study is consistent with these prior studies, and demonstrates that over 80% of smokers start smoking during adolescence. These findings underscore an urgent need for tobacco control strategies in rural China for adolescents and young adults who are at high risk for becoming addicted to nicotine.

As shown in other Chinese studies, smoking cigarettes was the most popular form of tobacco use in rural southwest China [10,25]. Furthermore, we found that consuming tobacco through hookah / water pipes was also common in the tobacco–cultivating, ethnically diverse, rural communities. Our findings indicate that cultural forces exert a strong effect on smoking habits, as has been demonstrated in previous studies [26].

Our study showed a high rate of exposure to SHS among current smokers, particularly in public places. The rate of exposure to SHS in public places observed in this study (81.1%) is much higher than the China national survey results of 2002 (67.0%) [3], as well as results from another Chinese study (72.7%) [27], indicating public places have become the worst places for SHS exposure in rural southwest China. Furthermore, our study showed that exposure to SHS among current smokers in households mostly occurred in front of children and pregnant women. While smoking among women in the study region was at a much lower level compared with men, over 75% of women were exposed to SHS, and the rate of exposure to SHS among women was higher than among men, indicating exposure to SHS is a serious health problem for women. The impact of SHS exposure on children’s and women’s health requires urgent attention. Evidence has shown that the implementation of comprehensive smoke-free policies decreases the exposure to SHS and its associated health hazards in non-smokers [28]. These results suggest it is essential to strengthen people’s awareness of tobacco hazards and to implement comprehensive smoke-free laws and legislations to protect people from SHS.

Our study found individuals who cultivated tobacco had higher prevalence rates of current smoking, exposure to SHS, and nicotine dependence than non-tobacco-cultivating people. This is possibly due to tobacco farmers having easier access to tobacco products. These results suggest tobacco cultivating status is an important consideration when developing tobacco control policies in rural China.

Ethnic minorities had a lower risk for exposure to SHS and nicotine dependence in this study. Genetic heritability and shared environmental influences have been reported to be related to cigarette smoking in a previous study [29]. This finding suggests that ethnicity is an important determinant for tobacco use.

Lower levels of education have been shown to be associated with higher risks of smoking, environmental tobacco exposure, nicotine dependence, and lower rates of quitting tobacco use, both in developed and developing countries [8,9,30]. In the present study, smoking, exposure to SHS, and nicotine dependence tended to be more prevalent in people who were less educated. Our result is in concordance with prior studies. Some previous Chinese studies also indicated an inverse association between education and smoking [2,11,12,18]. Our findings suggest community-based tobacco control efforts need to increase the knowledge related to harm of tobacco use among people with low levels of education.

While several western studies have demonstrated that people with lower incomes are more likely to consume tobacco [6-9], our study showed no association between individual income and smoking or exposure to SHS in the Chinese rural adults. However, it did show a positive relationship between individual income and nicotine dependence. A previous Chinese study reported that people with higher annual income were more likely to smoke than those with lower income [18]. The reasons for an inconsistent association between income and smoking rates in Chinese adult smokers are unknown.

A strength of our study is the large sample size and the high response rate (over 98%) in the community survey. A limitation of the study is that smoking was self-reported and exposure to SES was based on recall. The lack of validation of smoking status with nicotine testing may underestimate the prevalence of smoking.

## Conclusion

In conclusion, smoking and exposure to SHS are very prevalent in rural southwest China. The results of this study suggest that future tobacco control efforts and policies should focus more on people who cultivate tobacco, who are less educated, and who belong to the Han majority.

## Competing interests

The authors declare that they have no conflicts of interest.

## Authors' contributions

CL carried out the study and drafted and revised the manuscript. WX and HY participated in the design of the study. AG participated in the design of the study and helped to revise the manuscript. CW, XX, HJ, ZK, SY, and JF collected the data. All authors read and approved the final manuscript.

## Pre-publication history

The pre-publication history for this paper can be accessed here:

http://www.biomedcentral.com/1471-2458/12/842/prepub
